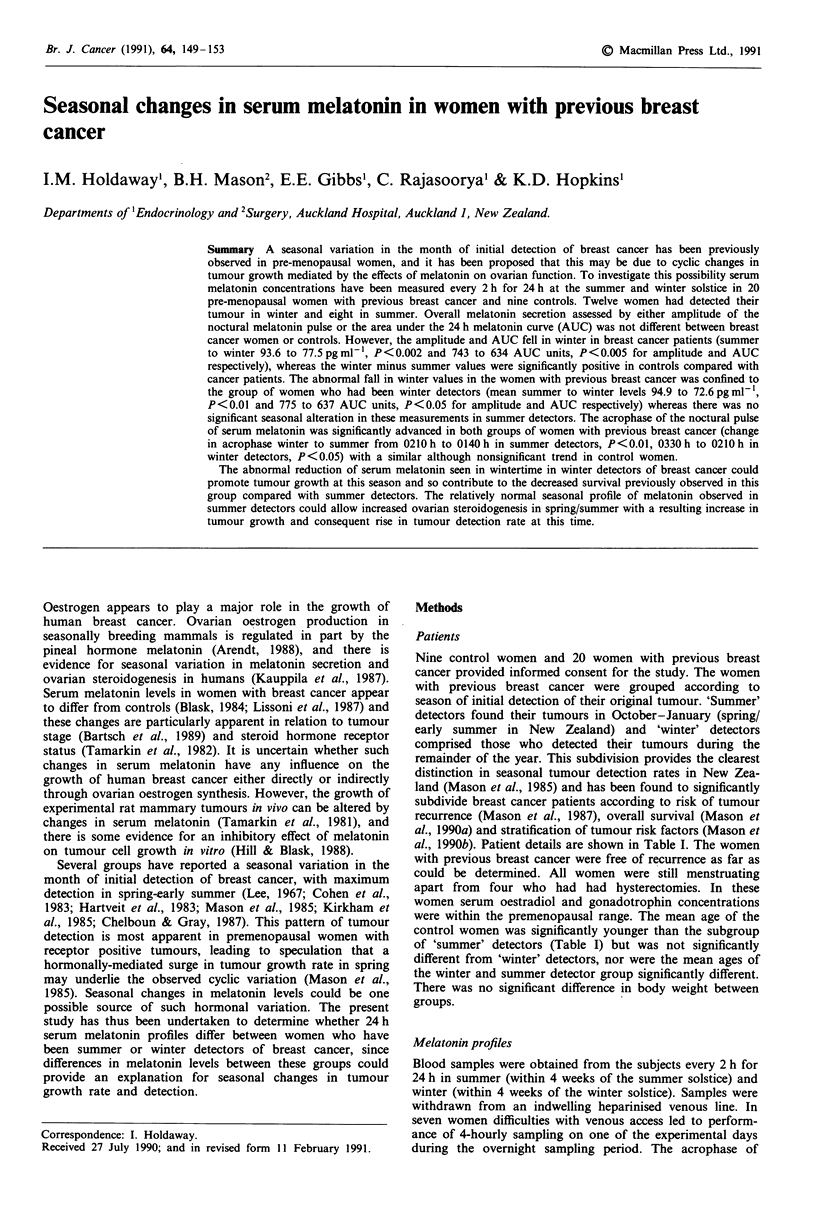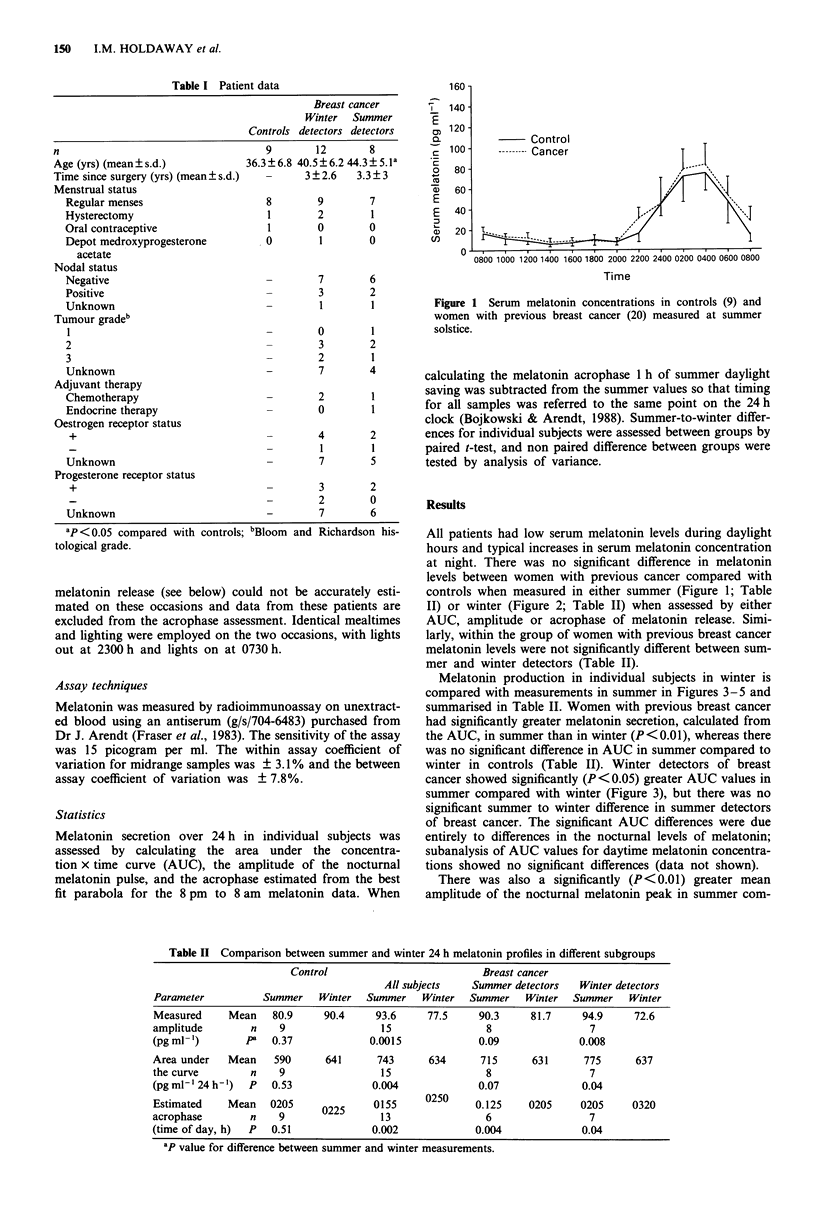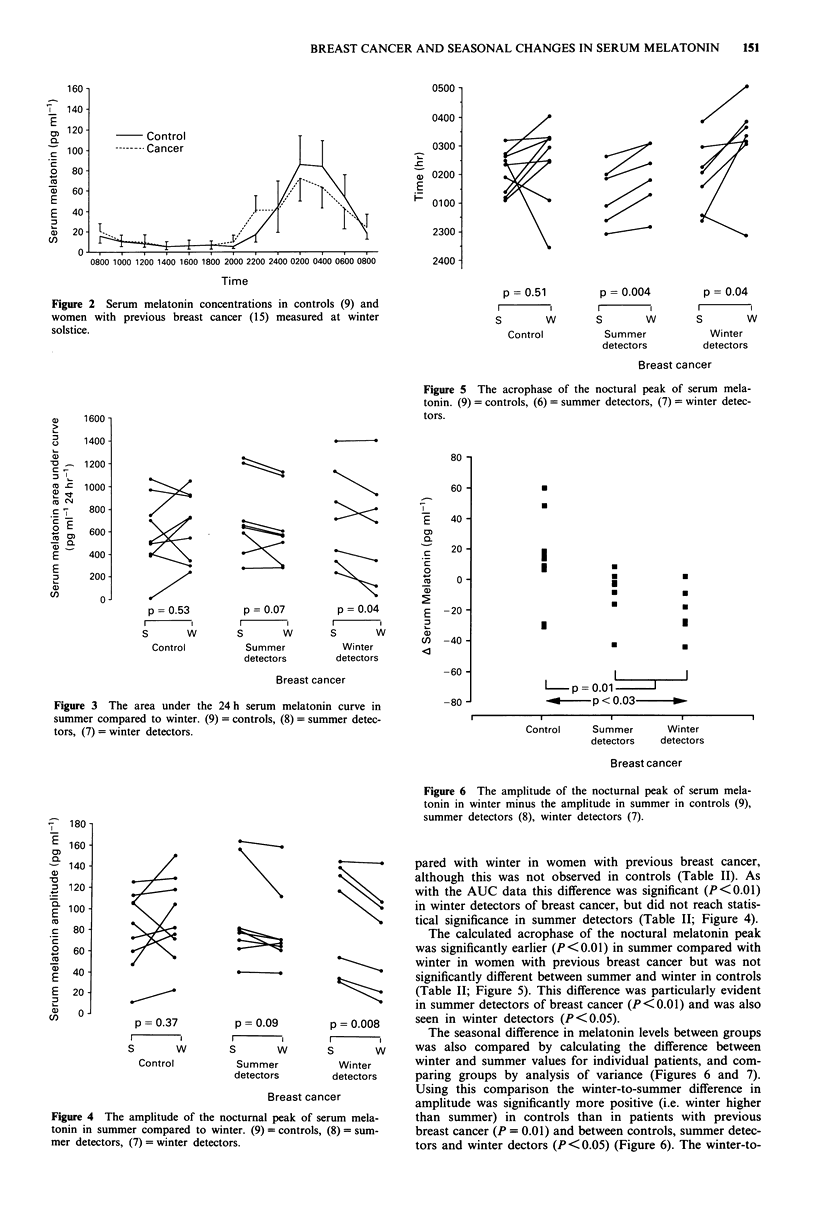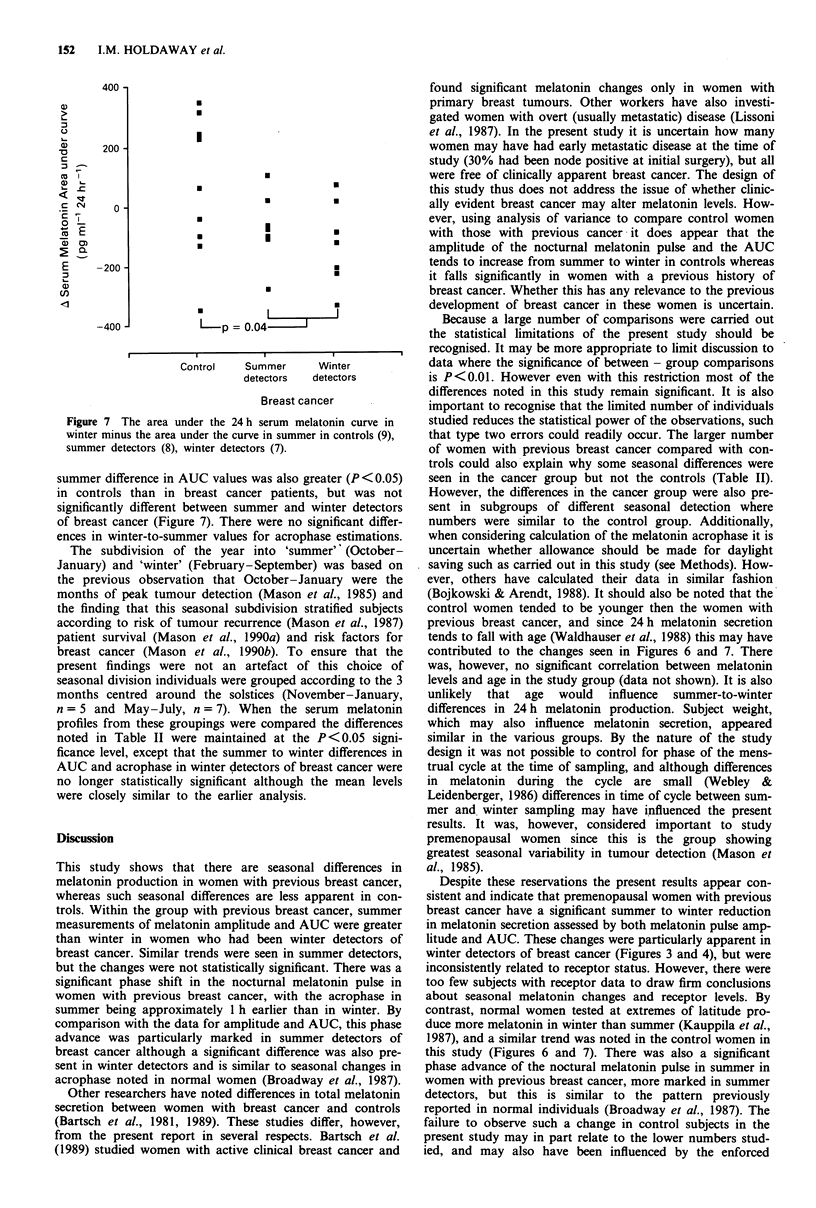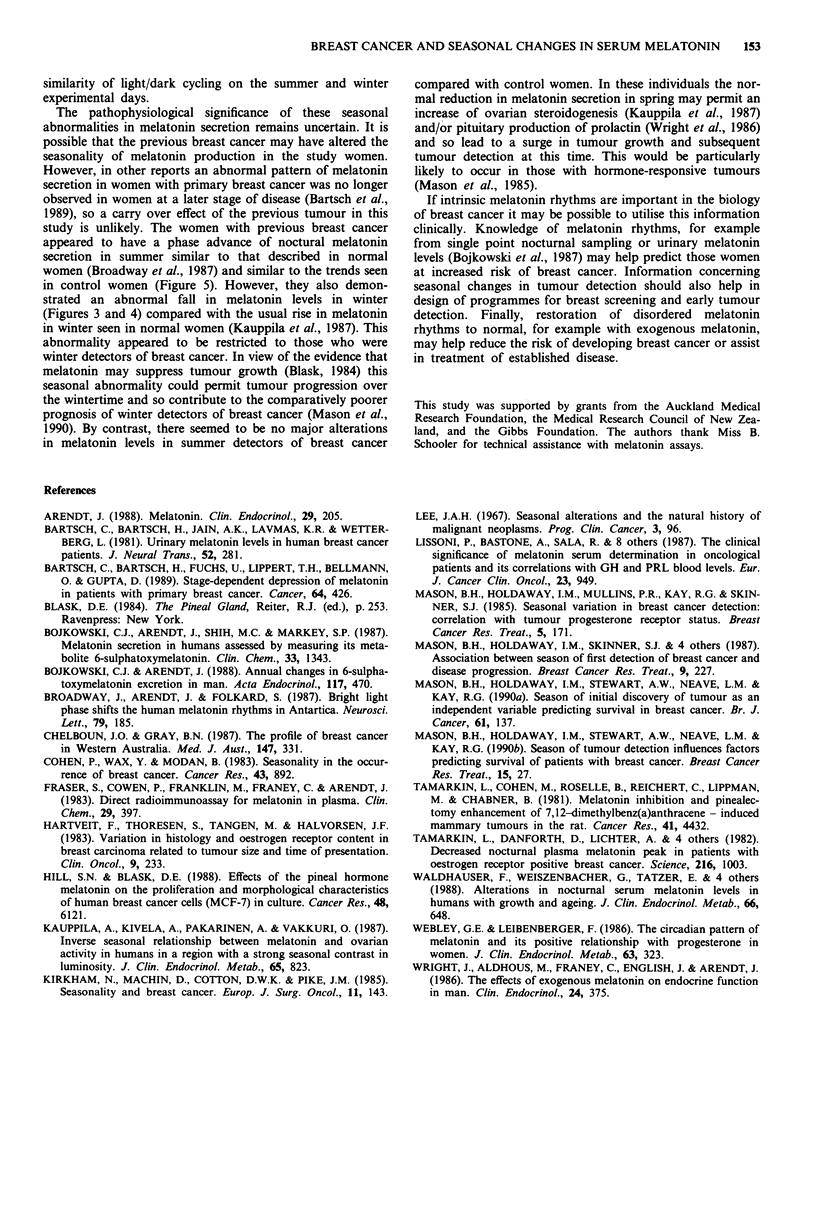# Seasonal changes in serum melatonin in women with previous breast cancer.

**DOI:** 10.1038/bjc.1991.259

**Published:** 1991-07

**Authors:** I. M. Holdaway, B. H. Mason, E. E. Gibbs, C. Rajasoorya, K. D. Hopkins

**Affiliations:** Department of Endocrinology, Auckland Hospital, New Zealand.

## Abstract

A seasonal variation in the month of initial detection of breast cancer has been previously observed in pre-menopausal women, and it has been proposed that this may be due to cyclic changes in tumour growth mediated by the effects of melatonin on ovarian function. To investigate this possibility serum melatonin concentrations have been measured every 2 h for 24 h at the summer and winter solstice in 20 pre-menopausal women with previous breast cancer and nine controls. Twelve women had detected their tumour in winter and eight in summer. Overall melatonin secretion assessed by either amplitude of the nocturnal melatonin pulse or the area under the 24 h melatonin curve (AUC) was not different between breast cancer women or controls. However, the amplitude and AUC fell in winter in breast cancer patients (summer to winter 93.6 to 77.5 pg ml-1, P less than 0.002 and 743 to 634 AUC units, P less than 0.005 for amplitude and AUC respectively), whereas the winter minus summer values were significantly positive in controls compared with cancer patients. The abnormal fall in winter values in the women with previous breast cancer was confined to the group of women who had been winter detectors (mean summer to winter levels 94.9 to 72.6 pg ml-1, P less than 0.01 and 775 to 637 AUC units, P less than 0.05 for amplitude and AUC respectively) whereas there was no significant seasonal alteration in these measurements in summer detectors. The acrophase of the nocturnal pulse of serum melatonin was significantly advanced in both groups of women with previous breast cancer (change in acrophase winter to summer from 0210 h to 0140 h in summer detectors, P less than 0.01, 0330 h to 0210 h in winter detectors, P less than 0.05) with a similar although nonsignificant trend in control women. The abnormal reduction of serum melatonin seen in wintertime in winter detectors of breast cancer could promote tumour growth at this season and so contribute to the decreased survival previously observed in this group compared with summer detectors. The relatively normal seasonal profile of melatonin observed in summer detectors could allow increased ovarian steroidogenesis in spring/summer with a resulting increase in tumour growth and consequent rise in tumour detection rate at this time.


Br. J. Cancer (1991), 64, 149-153 © Macmillan Press Ltd., 1991

### Seasonal changes in serum melatonin in women with previous breast cancer

I.M. Holdaway[1], B.H. Mason[2], E.E. Gibbs[1], C. Rajasoorya[1] & K.D. Hopkins[1]

*Departments of [1]Endocrinology and [2]Surgery, Auckland Hospital, Auckland 1, New Zealand.*

**Summary** A seasonal variation in the month of initial detection of breast cancer has been previously observed in pre-menopausal women, and it has been proposed that this may be due to cyclic changes in tumour growth mediated by the effects of melatonin on ovarian function. To investigate this possibility serum melatonin concentrations have been measured every 2 h for 24 h at the summer and winter solstice in 20 pre-menopausal women with previous breast cancer and nine controls. Twelve women had detected their tumour in winter and eight in summer. Overall melatonin secretion assessed by either amplitude of the noctural melatonin pulse or the area under the 24 h melatonin curve (AUC) was not different between breast cancer women or controls. However, the amplitude and AUC fell in winter in breast cancer patients (summer to winter 93.6 to 77.5 pg ml$^{-1}$, $P<0.002$ and 743 to 634 AUC units, $P<0.005$ for amplitude and AUC respectively), whereas the winter minus summer values were significantly positive in controls compared with cancer patients. The abnormal fall in winter values in the women with previous breast cancer was confined to the group of women who had been winter detectors (mean summer to winter levels 94.9 to 72.6 pg ml$^{-1}$, $P<0.01$ and 775 to 637 AUC units, $P<0.05$ for amplitude and AUC respectively) whereas there was no significant seasonal alteration in these measurements in summer detectors. The acrophase of the noctural pulse of serum melatonin was significantly advanced in both groups of women with previous breast cancer (change in acrophase winter to summer from 0210 h to 0140 h in summer detectors, $P<0.01$, 0330 h to 0210 h in winter detectors, $P<0.05$) with a similar although nonsignificant trend in control women.

The abnormal reduction of serum melatonin seen in wintertime in winter detectors of breast cancer could promote tumour growth at this season and so contribute to the decreased survival previously observed in this group compared with summer detectors. The relatively normal seasonal profile of melatonin observed in summer detectors could allow increased ovarian steroidogenesis in spring/summer with a resulting increase in tumour growth and consequent rise in tumour detection rate at this time.

Oestrogen appears to play a major role in the growth of human breast cancer. Ovarian oestrogen production in seasonally breeding mammals is regulated in part by the pineal hormone melatonin (Arendt, 1988), and there is evidence for seasonal variation in melatonin secretion and ovarian steroidogenesis in humans (Kauppila *et al.*, 1987). Serum melatonin levels in women with breast cancer appear to differ from controls (Blask, 1984; Lissoni *et al.*, 1987) and these changes are particularly apparent in relation to tumour stage (Bartsch *et al.*, 1989) and steroid hormone receptor status (Tamarkin *et al.*, 1982). It is uncertain whether such changes in serum melatonin have any influence on the growth of human breast cancer either directly or indirectly through ovarian oestrogen synthesis. However, the growth of experimental rat mammary tumours *in vivo* can be altered by changes in serum melatonin (Tamarkin *et al.*, 1981), and there is some evidence for an inhibitory effect of melatonin on tumour cell growth *in vitro* (Hill & Blask, 1988).

Several groups have reported a seasonal variation in the month of initial detection of breast cancer, with maximum detection in spring-early summer (Lee, 1967; Cohen *et al.*, 1983; Hartveit *et al.*, 1983; Mason *et al.*, 1985; Kirkham *et al.*, 1985; Chelboun & Gray, 1987). This pattern of tumour detection is most apparent in premenopausal women with receptor positive tumours, leading to speculation that a hormonally-mediated surge in tumour growth rate in spring may underlie the observed cyclic variation (Mason *et al.*, 1985). Seasonal changes in melatonin levels could be one possible source of such hormonal variation. The present study has thus been undertaken to determine whether 24 h serum melatonin profiles differ between women who have been summer or winter detectors of breast cancer, since differences in melatonin levels between these groups could provide an explanation for seasonal changes in tumour growth rate and detection.

Correspondence: I. Holdaway.
Received 27 July 1990; and in revised form 11 February 1991.

### Methods

### *Patients*

Nine control women and 20 women with previous breast cancer provided informed consent for the study. The women with previous breast cancer were grouped according to season of initial detection of their original tumour. 'Summer' detectors found their tumours in October–January (spring/early summer in New Zealand) and 'winter' detectors comprised those who detected their tumours during the remainder of the year. This subdivision provides the clearest distinction in seasonal tumour detection rates in New Zealand (Mason *et al.*, 1985) and has been found to significantly subdivide breast cancer patients according to risk of tumour recurrence (Mason *et al.*, 1987), overall survival (Mason *et al.*, 1990*a*) and stratification of tumour risk factors (Mason *et al.*, 1990*b*). Patient details are shown in Table I. The women with previous breast cancer were free of recurrence as far as could be determined. All women were still menstruating apart from four who had had hysterectomies. In these women serum oestradiol and gonadotrophin concentrations were within the premenopausal range. The mean age of the control women was significantly younger than the subgroup of 'summer' detectors (Table I) but was not significantly different from 'winter' detectors, nor were the mean ages of the winter and summer detector group significantly different. There was no significant difference in body weight between groups.

### *Melatonin profiles*

Blood samples were obtained from the subjects every 2 h for 24 h in summer (within 4 weeks of the summer solstice) and winter (within 4 weeks of the winter solstice). Samples were withdrawn from an indwelling heparinised venous line. In seven women difficulties with venous access led to performance of 4-hourly sampling on one of the experimental days during the overnight sampling period. The acrophase of

**Table I** Patient data

| | Controls | Breast cancer Winter detectors | Breast cancer Summer detectors |
|---|---|---|---|
| *n* | 9 | 12 | 8 |
| Age (yrs) (mean ± s.d.) | 36.3 ± 6.8 | 40.5 ± 6.2 | 44.3 ± 5.1[a] |
| Time since surgery (yrs) (mean ± s.d.) | – | 3 ± 2.6 | 3.3 ± 3 |
| Menstrual status | | | |
| Regular menses | 8 | 9 | 7 |
| Hysterectomy | 1 | 2 | 1 |
| Oral contraceptive | 1 | 0 | 0 |
| Depot medroxyprogesterone acetate | 0 | 1 | 0 |
| Nodal status | | | |
| Negative | – | 7 | 6 |
| Positive | – | 3 | 2 |
| Unknown | – | 1 | 1 |
| Tumour grade[b] | | | |
| 1 | – | 0 | 1 |
| 2 | – | 3 | 2 |
| 3 | – | 2 | 1 |
| Unknown | – | 7 | 4 |
| Adjuvant therapy | | | |
| Chemotherapy | – | 2 | 1 |
| Endocrine therapy | – | 0 | 1 |
| Oestrogen receptor status | | | |
| + | – | 4 | 2 |
| – | – | 1 | 1 |
| Unknown | – | 7 | 5 |
| Progesterone receptor status | | | |
| + | – | 3 | 2 |
| – | – | 2 | 0 |
| Unknown | – | 7 | 6 |

[a]$P < 0.05$ compared with controls; [b]Bloom and Richardson histological grade.

melatonin release (see below) could not be accurately estimated on these occasions and data from these patients are excluded from the acrophase assessment. Identical mealtimes and lighting were employed on the two occasions, with lights out at 2300 h and lights on at 0730 h.

*Assay techniques*

Melatonin was measured by radioimmunoassay on unextracted blood using an antiserum (g/s/704-6483) purchased from Dr J. Arendt (Fraser *et al.*, 1983). The sensitivity of the assay was 15 picogram per ml. The within assay coefficient of variation for midrange samples was ± 3.1% and the between assay coefficient of variation was ± 7.8%.

*Statistics*

Melatonin secretion over 24 h in individual subjects was assessed by calculating the area under the concentration × time curve (AUC), the amplitude of the nocturnal melatonin pulse, and the acrophase estimated from the best fit parabola for the 8 pm to 8 am melatonin data. When calculating the melatonin acrophase 1 h of summer daylight saving was subtracted from the summer values so that timing for all samples was referred to the same point on the 24 h clock (Bojkowski & Arendt, 1988). Summer-to-winter differences for individual subjects were assessed between groups by paired *t*-test, and non paired difference between groups were tested by analysis of variance.

**Figure 1** Serum melatonin concentrations in controls (9) and women with previous breast cancer (20) measured at summer solstice.

### Results

All patients had low serum melatonin levels during daylight hours and typical increases in serum melatonin concentration at night. There was no significant difference in melatonin levels between women with previous cancer compared with controls when measured in either summer (Figure 1; Table II) or winter (Figure 2; Table II) when assessed by either AUC, amplitude or acrophase of melatonin release. Similarly, within the group of women with previous breast cancer melatonin levels were not significantly different between summer and winter detectors (Table II).

Melatonin production in individual subjects in winter is compared with measurements in summer in Figures 3–5 and summarised in Table II. Women with previous breast cancer had significantly greater melatonin secretion, calculated from the AUC, in summer than in winter ($P < 0.01$), whereas there was no significant difference in AUC in summer compared to winter in controls (Table II). Winter detectors of breast cancer showed significantly ($P < 0.05$) greater AUC values in summer compared with winter (Figure 3), but there was no significant summer to winter difference in summer detectors of breast cancer. The significant AUC differences were due entirely to differences in the nocturnal levels of melatonin; subanalysis of AUC values for daytime melatonin concentrations showed no significant differences (data not shown).

There was also a significantly ($P < 0.01$) greater mean amplitude of the nocturnal melatonin peak in summer com-

**Table II** Comparison between summer and winter 24 h melatonin profiles in different subgroups

| Parameter | | Control Summer | Control Winter | Breast cancer All subjects Summer | All subjects Winter | Summer detectors Summer | Summer detectors Winter | Winter detectors Summer | Winter detectors Winter |
|---|---|---|---|---|---|---|---|---|---|
| Measured amplitude (pg ml$^{-1}$) | Mean | 80.9 | 90.4 | 93.6 | 77.5 | 90.3 | 81.7 | 94.9 | 72.6 |
| | *n* | 9 | | 15 | | 8 | | 7 | |
| | $P$[a] | 0.37 | | 0.0015 | | 0.09 | | 0.008 | |
| Area under the curve (pg ml$^{-1}$ 24 h$^{-1}$) | Mean | 590 | 641 | 743 | 634 | 715 | 631 | 775 | 637 |
| | *n* | 9 | | 15 | | 8 | | 7 | |
| | *P* | 0.53 | | 0.004 | | 0.07 | | 0.04 | |
| Estimated acrophase (time of day, h) | Mean | 0205 | 0225 | 0155 | 0250 | 0.125 | 0205 | 0205 | 0320 |
| | *n* | 9 | | 13 | | 6 | | 7 | |
| | *P* | 0.51 | | 0.002 | | 0.004 | | 0.04 | |

[a]*P* value for difference between summer and winter measurements.

**Figure 2** Serum melatonin concentrations in controls (9) and women with previous breast cancer (15) measured at winter solstice.

**Figure 3** The area under the 24 h serum melatonin curve in summer compared to winter. (9) = controls, (8) = summer detectors, (7) = winter detectors.

**Figure 4** The amplitude of the nocturnal peak of serum melatonin in summer compared to winter. (9) = controls, (8) = summer detectors, (7) = winter detectors.

**Figure 5** The acrophase of the noctural peak of serum melatonin. (9) = controls, (6) = summer detectors, (7) = winter detectors.

**Figure 6** The amplitude of the nocturnal peak of serum melatonin in winter minus the amplitude in summer in controls (9), summer detectors (8), winter detectors (7).

pared with winter in women with previous breast cancer, although this was not observed in controls (Table II). As with the AUC data this difference was significant ($P < 0.01$) in winter detectors of breast cancer, but did not reach statistical significance in summer detectors (Table II; Figure 4).

The calculated acrophase of the noctural melatonin peak was significantly earlier ($P < 0.01$) in summer compared with winter in women with previous breast cancer but was not significantly different between summer and winter in controls (Table II; Figure 5). This difference was particularly evident in summer detectors of breast cancer ($P < 0.01$) and was also seen in winter detectors ($P < 0.05$).

The seasonal difference in melatonin levels between groups was also compared by calculating the difference between winter and summer values for individual patients, and comparing groups by analysis of variance (Figures 6 and 7). Using this comparison the winter-to-summer difference in amplitude was significantly more positive (i.e. winter higher than summer) in controls than in patients with previous breast cancer ($P = 0.01$) and between controls, summer detectors and winter dectors ($P < 0.05$) (Figure 6). The winter-to-

**Figure 7** The area under the 24 h serum melatonin curve in winter minus the area under the curve in summer in controls (9), summer detectors (8), winter detectors (7).

summer difference in AUC values was also greater ($P<0.05$) in controls than in breast cancer patients, but was not significantly different between summer and winter detectors of breast cancer (Figure 7). There were no significant differences in winter-to-summer values for acrophase estimations.

The subdivision of the year into 'summer' (October–January) and 'winter' (February–September) was based on the previous observation that October–January were the months of peak tumour detection (Mason *et al.*, 1985) and the finding that this seasonal subdivision stratified subjects according to risk of tumour recurrence (Mason *et al.*, 1987) patient survival (Mason *et al.*, 1990*a*) and risk factors for breast cancer (Mason *et al.*, 1990*b*). To ensure that the present findings were not an artefact of this choice of seasonal division individuals were grouped according to the 3 months centred around the solstices (November–January, $n = 5$ and May–July, $n = 7$). When the serum melatonin profiles from these groupings were compared the differences noted in Table II were maintained at the $P<0.05$ significance level, except that the summer to winter differences in AUC and acrophase in winter detectors of breast cancer were no longer statistically significant although the mean levels were closely similar to the earlier analysis.

### Discussion

This study shows that there are seasonal differences in melatonin production in women with previous breast cancer, whereas such seasonal differences are less apparent in controls. Within the group with previous breast cancer, summer measurements of melatonin amplitude and AUC were greater than winter in women who had been winter detectors of breast cancer. Similar trends were seen in summer detectors, but the changes were not statistically significant. There was a significant phase shift in the nocturnal melatonin pulse in women with previous breast cancer, with the acrophase in summer being approximately 1 h earlier than in winter. By comparison with the data for amplitude and AUC, this phase advance was particularly marked in summer detectors of breast cancer although a significant difference was also present in winter detectors and is similar to seasonal changes in acrophase noted in normal women (Broadway *et al.*, 1987).

Other researchers have noted differences in total melatonin secretion between women with breast cancer and controls (Bartsch *et al.*, 1981, 1989). These studies differ, however, from the present report in several respects. Bartsch *et al.* (1989) studied women with active clinical breast cancer and found significant melatonin changes only in women with primary breast tumours. Other workers have also investigated women with overt (usually metastatic) disease (Lissoni *et al.*, 1987). In the present study it is uncertain how many women may have had early metastatic disease at the time of study (30% had been node positive at initial surgery), but all were free of clinically apparent breast cancer. The design of this study thus does not address the issue of whether clinically evident breast cancer may alter melatonin levels. However, using analysis of variance to compare control women with those with previous cancer it does appear that the amplitude of the nocturnal melatonin pulse and the AUC tends to increase from summer to winter in controls whereas it falls significantly in women with a previous history of breast cancer. Whether this has any relevance to the previous development of breast cancer in these women is uncertain.

Because a large number of comparisons were carried out the statistical limitations of the present study should be recognised. It may be more appropriate to limit discussion to data where the significance of between – group comparisons is $P<0.01$. However even with this restriction most of the differences noted in this study remain significant. It is also important to recognise that the limited number of individuals studied reduces the statistical power of the observations, such that type two errors could readily occur. The larger number of women with previous breast cancer compared with controls could also explain why some seasonal differences were seen in the cancer group but not the controls (Table II). However, the differences in the cancer group were also present in subgroups of different seasonal detection where numbers were similar to the control group. Additionally, when considering calculation of the melatonin acrophase it is uncertain whether allowance should be made for daylight saving such as carried out in this study (see Methods). However, others have calculated their data in similar fashion (Bojkowski & Arendt, 1988). It should also be noted that the control women tended to be younger then the women with previous breast cancer, and since 24 h melatonin secretion tends to fall with age (Waldhauser *et al.*, 1988) this may have contributed to the changes seen in Figures 6 and 7. There was, however, no significant correlation between melatonin levels and age in the study group (data not shown). It is also unlikely that age would influence summer-to-winter differences in 24 h melatonin production. Subject weight, which may also influence melatonin secretion, appeared similar in the various groups. By the nature of the study design it was not possible to control for phase of the menstrual cycle at the time of sampling, and although differences in melatonin during the cycle are small (Webley & Leidenberger, 1986) differences in time of cycle between summer and winter sampling may have influenced the present results. It was, however, considered important to study premenopausal women since this is the group showing greatest seasonal variability in tumour detection (Mason *et al.*, 1985).

Despite these reservations the present results appear consistent and indicate that premenopausal women with previous breast cancer have a significant summer to winter reduction in melatonin secretion assessed by both melatonin pulse amplitude and AUC. These changes were particularly apparent in winter detectors of breast cancer (Figures 3 and 4), but were inconsistently related to receptor status. However, there were too few subjects with receptor data to draw firm conclusions about seasonal melatonin changes and receptor levels. By contrast, normal women tested at extremes of latitude produce more melatonin in winter than summer (Kauppila *et al.*, 1987), and a similar trend was noted in the control women in this study (Figures 6 and 7). There was also a significant phase advance of the noctural melatonin pulse in summer in women with previous breast cancer, more marked in summer detectors, but this is similar to the pattern previously reported in normal individuals (Broadway *et al.*, 1987). The failure to observe such a change in control subjects in the present study may in part relate to the lower numbers studied, and may also have been influenced by the enforced similarity of light/dark cycling on the summer and winter experimental days.

The pathophysiological significance of these seasonal abnormalities in melatonin secretion remains uncertain. It is possible that the previous breast cancer may have altered the seasonality of melatonin production in the study women. However, in other reports an abnormal pattern of melatonin secretion in women with primary breast cancer was no longer observed in women at a later stage of disease (Bartsch *et al.*, 1989), so a carry over effect of the previous tumour in this study is unlikely. The women with previous breast cancer appeared to have a phase advance of noctural melatonin secretion in summer similar to that described in normal women (Broadway *et al.*, 1987) and similar to the trends seen in control women (Figure 5). However, they also demonstrated an abnormal fall in melatonin levels in winter (Figures 3 and 4) compared with the usual rise in melatonin in winter seen in normal women (Kauppila *et al.*, 1987). This abnormality appeared to be restricted to those who were winter detectors of breast cancer. In view of the evidence that melatonin may suppress tumour growth (Blask, 1984) this seasonal abnormality could permit tumour progression over the wintertime and so contribute to the comparatively poorer prognosis of winter detectors of breast cancer (Mason *et al.*, 1990). By contrast, there seemed to be no major alterations in melatonin levels in summer detectors of breast cancer compared with control women. In these individuals the normal reduction in melatonin secretion in spring may permit an increase of ovarian steroidogenesis (Kauppila *et al.*, 1987) and/or pituitary production of prolactin (Wright *et al.*, 1986) and so lead to a surge in tumour growth and subsequent tumour detection at this time. This would be particularly likely to occur in those with hormone-responsive tumours (Mason *et al.*, 1985).

If intrinsic melatonin rhythms are important in the biology of breast cancer it may be possible to utilise this information clinically. Knowledge of melatonin rhythms, for example from single point nocturnal sampling or urinary melatonin levels (Bojkowski *et al.*, 1987) may help predict those women at increased risk of breast cancer. Information concerning seasonal changes in tumour detection should also help in design of programmes for breast screening and early tumour detection. Finally, restoration of disordered melatonin rhythms to normal, for example with exogenous melatonin, may help reduce the risk of developing breast cancer or assist in treatment of established disease.

This study was supported by grants from the Auckland Medical Research Foundation, the Medical Research Council of New Zealand, and the Gibbs Foundation. The authors thank Miss B. Schooler for technical assistance with melatonin assays.

### References

ARENDT, J. (1988). Melatonin. *Clin. Endocrinol.*, **29**, 205.

BARTSCH, C., BARTSCH, H., JAIN, A.K., LAVMAS, K.R. & WETTERBERG, L. (1981). Urinary melatonin levels in human breast cancer patients. *J. Neural Trans.*, **52**, 281.

BARTSCH, C., BARTSCH, H., FUCHS, U., LIPPERT, T.H., BELLMANN, O. & GUPTA, D. (1989). Stage-dependent depression of melatonin in patients with primary breast cancer. *Cancer*, **64**, 426.

BLASK, D.E. (1984). *The Pineal Gland*, Reiter, R.J. (ed.), p. 253. Ravenpress: New York.

BOJKOWSKI, C.J., ARENDT, J., SHIH, M.C. & MARKEY, S.P. (1987). Melatonin secretion in humans assessed by measuring its metabolite 6-sulphatoxymelatonin. *Clin. Chem.*, **33**, 1343.

BOJKOWSKI, C.J. & ARENDT, J. (1988). Annual changes in 6-sulphatoxymelatonin excretion in man. *Acta Endocrinol.*, **117**, 470.

BROADWAY, J., ARENDT, J. & FOLKARD, S. (1987). Bright light phase shifts the human melatonin rhythms in Antartica. *Neurosci. Lett.*, **79**, 185.

CHELBOUN, J.O. & GRAY, B.N. (1987). The profile of breast cancer in Western Australia. *Med. J. Aust.*, **147**, 331.

COHEN, P., WAX, Y. & MODAN, B. (1983). Seasonality in the occurrence of breast cancer. *Cancer Res.*, **43**, 892.

FRASER, S., COWEN, P., FRANKLIN, M., FRANEY, C. & ARENDT, J. (1983). Direct radioimmunoassay for melatonin in plasma. *Clin. Chem.*, **29**, 397.

HARTVEIT, F., THORESEN, S., TANGEN, M. & HALVORSEN, J.F. (1983). Variation in histology and oestrogen receptor content in breast carcinoma related to tumour size and time of presentation. *Clin. Oncol.*, **9**, 233.

HILL, S.N. & BLASK, D.E. (1988). Effects of the pineal hormone melatonin on the proliferation and morphological characteristics of human breast cancer cells (MCF-7) in culture. *Cancer Res.*, **48**, 6121.

KAUPPILA, A., KIVELA, A., PAKARINEN, A. & VAKKURI, O. (1987). Inverse seasonal relationship between melatonin and ovarian activity in humans in a region with a strong seasonal contrast in luminosity. *J. Clin. Endocrinol. Metab.*, **65**, 823.

KIRKHAM, N., MACHIN, D., COTTON, D.W.K. & PIKE, J.M. (1985). Seasonality and breast cancer. *Europ. J. Surg. Oncol.*, **11**, 143.

LEE, J.A.H. (1967). Seasonal alterations and the natural history of malignant neoplasms. *Prog. Clin. Cancer*, **3**, 96.

LISSONI, P., BASTONE, A., SALA, R. & 8 others (1987). The clinical significance of melatonin serum determination in oncological patients and its correlations with GH and PRL blood levels. *Eur. J. Cancer Clin. Oncol.*, **23**, 949.

MASON, B.H., HOLDAWAY, I.M., MULLINS, P.R., KAY, R.G. & SKINNER, S.J. (1985). Seasonal variation in breast cancer detection: correlation with tumour progesterone receptor status. *Breast Cancer Res. Treat.*, **5**, 171.

MASON, B.H., HOLDAWAY, I.M., SKINNER, S.J. & 4 others (1987). Association between season of first detection of breast cancer and disease progression. *Breast Cancer Res. Treat.*, **9**, 227.

MASON, B.H., HOLDAWAY, I.M., STEWART, A.W., NEAVE, L.M. & KAY, R.G. (1990*a*). Season of initial discovery of tumour as an independent variable predicting survival in breast cancer. *Br. J. Cancer*, **61**, 137.

MASON, B.H., HOLDAWAY, I.M., STEWART, A.W., NEAVE, L.M. & KAY, R.G. (1990*b*). Season of tumour detection influences factors predicting survival of patients with breast cancer. *Breast Cancer Res. Treat.*, **15**, 27.

TAMARKIN, L., COHEN, M., ROSELLE, B., REICHERT, C., LIPPMAN, M. & CHABNER, B. (1981). Melatonin inhibition and pinealectomy enhancement of 7,12–dimethylbenz(a)anthracene – induced mammary tumours in the rat. *Cancer Res.*, **41**, 4432.

TAMARKIN, L., DANFORTH, D., LICHTER, A. & 4 others (1982). Decreased nocturnal plasma melatonin peak in patients with oestrogen receptor positive breast cancer. *Science*, **216**, 1003.

WALDHAUSER, F., WEISZENBACHER, G., TATZER, E. & 4 others (1988). Alterations in nocturnal serum melatonin levels in humans with growth and ageing. *J. Clin. Endocrinol. Metab.*, **66**, 648.

WEBLEY, G.E. & LEIBENBERGER, F. (1986). The circadian pattern of melatonin and its positive relationship with progesterone in women. *J. Clin. Endocrinol. Metab.*, **63**, 323.

WRIGHT, J., ALDHOUS, M., FRANEY, C., ENGLISH, J. & ARENDT, J. (1986). The effects of exogenous melatonin on endocrine function in man. *Clin. Endocrinol.*, **24**, 375.